# Mono-6-Deoxy-6-Aminopropylamino-*β*-Cyclodextrin on Ag-Embedded SiO_2_ Nanoparticle as a Selectively Capturing Ligand to Flavonoids

**DOI:** 10.3390/nano9101349

**Published:** 2019-09-20

**Authors:** Eunil Hahm, Eun Ji Kang, Xuan-Hung Pham, Daham Jeong, Dae Hong Jeong, Seunho Jung, Bong-Hyun Jun

**Affiliations:** 1 Department of Bioscience and Biotechnology, Konkuk University, Seoul 05029, Korea; greenice@konkuk.ac.kr (E.H.); ejkang@konkuk.ac.kr (E.J.K.); phamricky@gmail.com (X.-H.P.); amir@konkuk.ac.kr (D.J.); shjung@konkuk.ac.kr (S.J.); 2 Department of Chemistry Education and Center for Educational Research, Seoul National University, Seoul 08826, Korea; jeongdh@snu.ac.kr

**Keywords:** mono-6-deoxy-6-aminopropylamino-*β*-cyclodextrin, pr-*β*-CD, flavonoid, selectivity, Ag-embedded SiO_2_, SERS

## Abstract

It has been increasingly important to develop a highly sensitive and selective technique that is easy to handle in detecting levels of beneficial or hazardous analytes in trace quantity. In this study, mono-6-deoxy-6-aminopropylamino-*β*-cyclodextrin (pr-*β*-CD)-functionalized silver-assembled silica nanoparticles (SiO_2_@Ag@pr-*β*-CD) for flavonoid detection were successfully prepared. The presence of pr-*β*-CD on the surface of SiO_2_@Ag enhanced the selectivity in capturing quercetin and myricetin among other similar materials (naringenin and apigenin). In addition, SiO_2_@Ag@pr-*β*-CD was able to detect quercetin corresponding to a limit of detection (LOD) as low as 0.55 ppm. The relationship between the Raman intensity of SiO_2_@Ag@pr-*β*-CD and the logarithm of the Que concentration obeyed linearity in the range 3.4–33.8 ppm (R^2^ = 0.997). The results indicate that SiO_2_@Ag@pr-*β*-CD is a promising material for immediately analyzing samples that demand high sensitivity and selectivity of detection.

## 1. Introduction

Nowadays, it is necessary to develop convenient techniques with high sensitivity and selectivity in detecting beneficial or hazardous trace level analytes. The development of high sensitivity detection methods based on nanomaterials has been supported by recent advancements of nanotechnology. In particular, nanotech-based surface-enhanced Raman scattering (SERS) spectroscopy has been an attractive method as it enables an ultra-fast and universal analytical technique for various materials, such as flavonoids, polycyclic aromatic hydrocarbons (PAHs), and endocrine disruptor chemicals (EDCs) [1,2,3,4,5,6,7]. Most research has focused on direct detection methods whereby analyzing the SERS peaks of target molecules located near or on the surface of metal nanoparticles (NPs) allows for high sensitivity and a strong multiplexing ability [8,9,10]. However, problems exist due to the differences in the affinity of functional groups to the metal surface, which limits the application of SERS for target detection with the direct method [11]. To increase the affinity of specific targets, ligands and small organic molecules have been introduced on the surface of metals [1,2,3,5,12].

Cyclodextrin (CD) has cavities with a hydrophobic interior and hydrophilic exterior and thus is capable of forming inclusion compounds with guest molecules [13]. The formation of inclusion compounds supports and stabilizes the properties of the guest materials, which has made CD a popular industrial and analytical material. For example, potentiometric sensor-incorporated neutral *β*-CD was able to enhance the sensitivity and selectivity of electrodes in ion molecules, especially naproxen [14]. *β*-CD attached to a lactone ring on the primary side of the CD also showed a large affinity to bile acids by the interaction between the chromophore unit and the guest molecule in the complex [15]. A forchlorfenuron-imprinted polymer utilizing *β*-CD as a monomer had better recognition and reversibility to forchlorfenuron [16]. In addition, depending on the attached substance, *β*-CD has made it possible to identify a neutral chiral agent for chiral neutral enantiomers [17]. As a result, CD has become a highly valuable material not only for developing novel materials through its modification but also for expanding applications according to the characteristics of guest materials due to its superb applicability.

Recently, our group reported the successful synthesis of silver nanoparticle assembled silica nanostructures using octylamine in ethylene glycol in the presence of polyvinylpyrrolidone [11,18,19,20,21]. The presence of the bumpy silver structure on the surface of silica creates a reliable SERS-active nanomaterial that produces stable and strong SERS signals because of the hotspot structure [22]. Ag-embedded silica nanostructures also produced a highly reproducible SERS signals and could be scaled up [21]. As a result, highly sensitive detection of polycyclic aromatic hydrocarbons (PAHs), flavonoids, and the like was successfully developed [23,24,25,26,27]. Subsequently, *β*-CD dimer was attached on the surface of Ag-embedded SiO_2_ NPs to develop the detection of PAHs in our group. The bridged dimeric *β*-CD and assembled structure showed sensitive and multiplex detection of PAHs in SERS measurement [26]. Also, the interaction of the modified form of *β*-CD with flavonoids was evidenced by comparing UV absorption [27]. Among the *β*-CD derivatives, ethylenediamine *β*-CD showed an outstanding complex-forming ability with flavonoid. We successfully developed a highly sensitive method in detecting flavonoid molecules; however, the function of ligands that can selectively detect the targets, especially those with similar structures, has not yet been demonstrated.

In this study, we investigated the critical role of the ligand in detecting materials with similar structures. Mono-6-deoxy-6-aminopropylamino-*β*-cyclodextrin (pr-*β*-CD) was modified as a ligand on the surface of SiO_2_@Ag. The modified *β*-CD was used as a SERS substrate for the detection of flavonoids, namely quercetin (Que) and myricetin (Myr), and resulted in a high selectivity for these flavonoids. SiO_2_@Ag@pr-*β*-CD also provided a reliable technique for quantitative detection of Que.

## 2. Materials and Methods

### 2.1. Chemical and Materials

Tetraethylorthosilicate (TEOS), 3-mercaptopropyl trimethoxysilane (MPTS), ethylene glycol (EG), silver nitrate (AgNO_3_), octylamine (OA), ethyl alcohol (EtOH, 99.5% and 95%), 1-(p-toluenesulfonyl) imidazole, sodium hydroxide (NaOH), ammonium chloride (NH_4_Cl), acetone, propane-1, 3-diamine, *β*-cyclodextin, and polyvinylpyrrolidone (PVP, MW 40,000) were purchased from Sigma-Aldrich (St. Louis, MO, USA) and used without further purification. Aqueous ammonium hydroxide (NH_4_OH, 27%) was purchased from Daejung (Siheung, Korea). Water was purified using a Direct-Q Millipore water purification system (SAM WOO S&T Co., Ltd., Seoul, Korea). Tosyl *β*-CD was obtained from microbial Carbohydrate Resource Bank (Seoul, Korea).

### 2.2. Preparation of SiO_2_@Ag

SiO_2_ NPs were synthesized by mixing TEOS (1.6 mL) in absolute EtOH (40 mL) and NH_4_OH (3 mL) utilizing the Stöber method [28]. The mixture was stirred vigorously for 20 h at room temperature (RT), and then excess reagents were removed by centrifuging and washing five times with EtOH. MPTS (200 µL) and NH_4_OH (40 µL) were mixed with SiO_2_ NPs (200 mg) in EtOH (8 mL) to introduce thiol groups on the surface of SiO_2_ NPs to attach the Ag NPs. The solution was stirred vigorously for 6 h and then centrifuged. The thiolated SiO_2_ NPs were washed five times with EtOH. Ag NPs with bumpy surfaces were introduced to the surface of SiO_2_ NPs by reducing silver nitrate (AgNO_3_) with octylamine (OA). Briefly, the thiolated SiO_2_ NPs (30 mg) were mixed with 50 mL of EG-containing PVP (5 mg) and AgNO_3_ (26 mg), followed by an addition of OA solution (41.4 µL) to obtain 8 mM OA [26]. One hour later, the solution was centrifuged, and the SiO_2_@Ag NPs were washed five times with EtOH to remove excess reagent. SiO_2_@Ag was dispersed in EtOH (30 mL).

### 2.3. Preparation of Pr-β-CD

The pr-*β*-CD was synthesized according to the previously reported method [29]. The *β*-CD (10 g) in water (250 mL) was reacted with 1-(p-toluenesulfonyl) imidazole (6 g) for 6 h. Sodium hydroxide (4.5 g) in water (12.5 mL) was mixed slowly over 20 min with the solution. The unreacted 1-(p-toluenesulfonyl) imidazole was removed by filtration after 10 min. To quench the reaction, ammonium chloride (NH_4_Cl, 12.05 g) was added to the reaction mixture. Tosyl *β*-CD was crystallized from the solution by evaporation. After filtering the suspension, the tosyl *β*-CD was washed with acetone and cold water twice and dried in vacuum. After dissolving the tosyl *β*-CD (1.5 g) in propane-1,3-diamine (5 mL) under N_2_, the solution was stirred at 50 °C for 12 h and was cooled to the RT. The cooled compound was precipitated using EtOH (200 mL). This step of using propane-1,3-diamin and ethanol was done three times and purified by flash column chromatography. The yield was 520 mg (55.7%). In the ^1^H NMR spectrum of pr-*β*-CD, the H1 to H6 protons of the glucopyranose units were exhibited at 5.07, 3.66, 3.85, 3.56, 3.97, and 3.86 ppm, respectively. The H7, H8, and H9 protons of the propane-1, 3-diamine were exhibited at 2.92, 1.78, and 2.66 ppm, respectively. Also, the 7:2 integral ratios of H1 to H9 protons confirmed one amino group of propylene-1, 3-diamine conjugated to *β*-CD. In the ^13^C NMR spectrum of pr-*β*-CD, the chemical shift of C1–C6 was assigned at 101.84, 72.09, 73.10, 81.15, 72.09, and 60.31 ppm, respectively. The C7, C8, and C9 were assigned at 38.10, 28.07, and 38.10 ppm, respectively. The properties of the synthesized pr-*β*-CD have been reported more specifically in another paper [29].

### 2.4. Preparation of Pr-β-CD Functionalized SiO_2_@Ag

Pr-*β*-CD-functionalized SiO_2_@Ag was prepared by mixing pr-*β*-CD in distilled water (500 μL, 2 mM) with EtOH (500 μL) containing SiO_2_@Ag (1 mg) for 1 h. The mixture was centrifuged and washed five times with EtOH, and the resulting pr-*β*-CD-functionalized SiO_2_@Ag was dispersed in EtOH (1 mL).

### 2.5. Incubation of SiO_2_@Ag@ Pr-β-CD with Flavonoids

Briefly, the SiO_2_@Ag@pr-*β*-CD (0.1 mg) was incubated with 10 ^−8^ mol of flavonoids (Que, Api, Myr, and Nar) for 12 h. The colloids were centrifuged at 12,000 rpm for 10 min and washed three times by EtOH. For comparison, SiO_2_@Ag (0.1 mg) was also incubated with the flavonoids as a control sample.

### 2.6. Characterization of Pr-β-CD Functionalized SiO_2_@Ag by TEM and UV-Visible Absorption Spectroscopy

A TEM microscope (LIBRA 120, Carl Zeiss, Oberkochen, Baden-Württemberg, Germany) was used to obtain TEM images. The size of NPs was analyzed by digitalized measurement using Image J software (National Institutes of Health, Bethesda, MD, USA), and the average size was calculated based on analysis of at least 100 NPs. An Optizen POP spectrophotometer (Mecasys Co., Ltd., Daejeon, Korea) was used to obtain UV-visible absorption spectra.

### 2.7. Raman Measurement

To evaluate the SERS spectra of the pr-*β*-CD functionalized SiO_2_@Ag reacted with flavonoids, the samples in solution state were measured with a capillary tube using a DXR™ Raman Microscope (Thermo Fisher Scientific, Madison, WI, USA). The SERS signals were collected with a backscattering geometry using a ×10 objective lens. A 532-nm diode-pumped solid-state laser was used as a photo-excitation source with a 5 mW laser power at the sample with a spot size of ~2 µm. All SERS spectra were integrated for 2 s, and the SERS signals of the samples were collected at random positions. Because no difference was observed between the integrated intensity and the height of the peak, we used the height of the peak for convenient calculation.

## 3. Results and Discussion

### Synthesis and Characterization of SiO_2_@Ag@Pr-β-CD

**Scheme 1:**
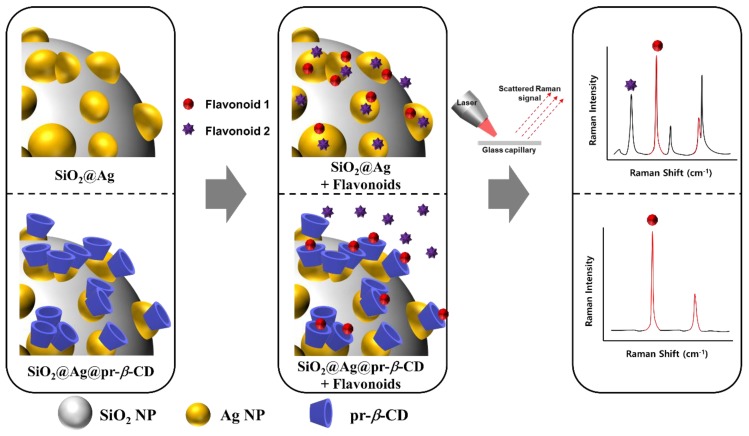
Schematic illustration of the effects of pr-*β*-CD on the surface of SiO_2_@Ag in selective flavonoid detection.

To investigate the selectivity of pr-*β*-CD as a flavonoid capturing ligand, SiO_2_@Ag was prepared as a substrate for pr-*β*-CD. SiO_2_ NP was used as a core material, capitulating its ease of surface modification. Ag NP on the SiO_2_ was used as a substrate to allow the interaction of ligands to capture target molecules, and also functioned as an enhancer of the SERS signal of the target. Scheme 1 illustrates the mechanism of pr-*β*-CD on SiO_2_@Ag as a selective flavonoid-capturing material. Without pr-*β*-CD, various kinds of flavonoids can attach to the SiO_2_@Ag surface, whereas the presence of pr-*β*-CD on the SiO_2_@Ag surface can induce selective capturing of certain flavonoids. The SERS enhance factor (EF) of the Ag-assembled silica structure is expected to be 1.0 × 10^5^. Despite the relatively low SERS EF, the signal that came from many target molecules at the active site is strong with low SERS signal fluctuation duo to the homogenous structure [21,30].

**Figure 1:**
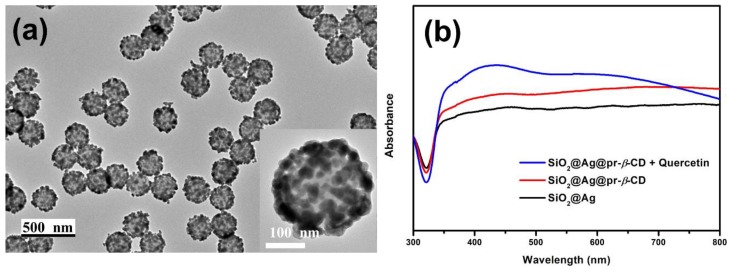
(**a**) TEM images of single SiO_2_@Ag (inlet) and distribution of SiO_2_@Ag in EtOH. (**b**) UV-visible absorption spectra of nanoparticles (NPs) at each step of the synthesis and that of SiO_2_@Ag@pr-*β*-CD added quercetin.

SiO_2_@Ag (Figure 1a) was synthesized according to the previous report [26]. The SiO_2_@Ag has a spherical shape, uniformly sized at 231.68 ± 8.49 nm, and pr-*β*-CD was introduced on the surface of the SiO_2_@Ag as a ligand for capturing specific targets. The introduction of the pr-*β*-CD on the surface was identified using the presence of a peak at 1290 cm^−1^, which is assigned to N-H stretching vibration, confirmed by the attenuated total reflection-Fourier transform infrared spectroscopy (ATR-FTIR) (Figure S1).

UV-visible absorption spectra were measured at each step of the synthesis to observe the optical properties of our materials (Figure 1b). SiO_2_@Ag (black line) showed a broad band between 350 and 800 nm due to the presence of Ag NPs on the silica NPs [19]. After introducing pr-*β*-CD on the surface of the Ag NPs, the SiO_2_@Ag@pr-*β*-CD (red line) showed a similar spectrum with that of the SiO_2_@Ag but with a slight increase in absorbance. Que was chosen as a model for the flavonoid in our study. The UV-visible absorption spectrum of SiO_2_@Ag@pr-*β*-CD mixed with Que (blue line) showed an overall increase, especially at the 350–450 nm range. In fact, Que demonstrated two absorption bands of flavonoid compounds in the ranges of 300 to 500 nm and 240 to 285 nm. Api, Myr, and Nar exhibited similar behavior in their absorption bands when they were incubated with SiO_2_@Ag@pr-*β*-CD (Figure S2). The results demonstrate that the presence of Que on the surface of SiO_2_@Ag@pr-*β*-CD may have brought a synergistic effect on the extinction co-efficiency to visible light, which resulted in an increased intensity of the absorbance bands in our material.

**Figure 2:**
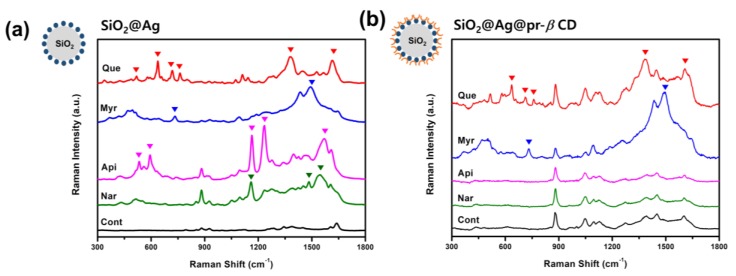
(**a**) Raman spectra of SiO_2_@Ag with four flavonoids—quercetin (Que), myricetin (Myr), apigenin (Api), and naringenin (Nar). (**b**) Raman spectra of SiO_2_@Ag@pr-*β*-CD with the four flavonoids.

To confirm the effect of pr-*β*-CD on the surface of SiO_2_@Ag, Raman spectra of SiO_2_@Ag@pr-*β*-CD and SiO_2_@Ag reacted with flavonoids were investigated. Four flavonoids with a similar structure—i.e., Que, Myr, Api, and Nar—were used as target molecules. As shown in Figure 2a, the Raman spectra of the SiO_2_@Ag reacted with these flavonoids showed their intrinsic characteristic bands. Que exhibited unique peaks at 637, 718, 762, 1380, and 1614 cm^−1^, while Myr showed unique peaks at 734 and 1495 cm^−1^. The characteristic bands of Api were at 533, 594, 1164, 1235, and 1570 cm^−1^, and those of Nar were at 1160, 1484, and 1545 cm^−1^. On the other hand, the SiO_2_@Ag@pr-*β*-CD incubated with four flavonoids showed the characteristics of SERS bands of Que and Myr (Figure 2b). More specifically, Que-incubated SiO_2_@Ag@pr-*β*-CD and Myr-incubated SiO_2_@Ag@pr-*β*-CD showed their unique peaks, regardless of the presence or absence of pr-*β*-CDs on the SiO_2_@Ag surface, although the shapes were slightly different. However, Api- and Nar-incubated SiO_2_@Ag@pr-*β*-CD were not able to show their characteristic peaks, and their SERS bands were similar to those of the SiO_2_@Ag@pr-*β*-CD, as demonstrated in Figure 2b. Not only the number of OH groups but also the position of OH groups on the structure of flavonoids are very important for the formation of the host–guest complexes between *β*-CD ligand and flavonoids. The position and number of OHs in the flavonoids affects the value of the stability constant associated with the *β*-CD [31]. It is well known that Que forms the most stable host–guest complex with *β*-CD compared to other three flavonoids [32,33,34,35]. In particular, Myr has a lower stability constant with *β*-CDs than that of Api. However, Myr has many OH groups, and it is thought to be more attractive to *β*-CDs by OH groups and hydrogen bonds and van der Waals attraction on the *β*-CD surface in ethanol solution [36,37]. The findings imply that Api and Nar could not be absorbed on the surface of the SiO_2_@Ag@pr-*β*-CD and indicate that the presence of pr-*β*-CDs on the surface of SiO_2_@Ag could selectively capture specific flavonoids among others with a similar structure.

**Figure 3:**
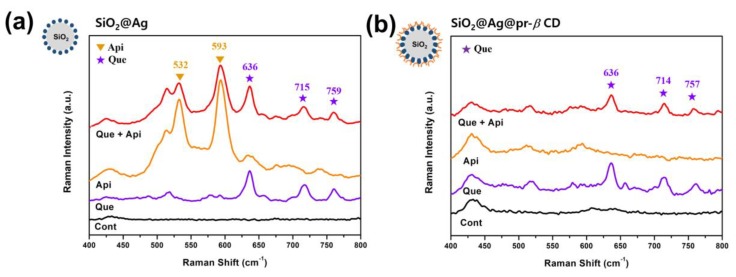
(**a**) The Raman intensity of SiO_2_@Ag-added mixture of apigenin (Api) and quercetin (Que). (**b**) The Raman intensity of SiO_2_@Ag@pr-*β*-CD-added mixture of Api and Que.

Next, we investigated the selectivity of the SiO_2_@Ag@pr-*β*-CDs for flavonoids. Three solutions were prepared (Api solution, Que solution, and their mixture solution), with each having an equal concentration. The solutions were mixed with SiO_2_@Ag and SiO_2_@Ag@pr-*β*-CDs, respectively, and the Raman spectra were measured. As can be seen in Figure 3, the SiO_2_@Ag in the mixture of Api and Que showed distinctive bands at 532, 593, 636 715, and 759 cm^−1^. The Raman peaks at 532 and 593 cm^−1^ were assigned to the characteristic peaks of Api, and the peaks at 636, 715, and 759 cm^−1^ to those of Que. In terms of SiO_2_@Ag@pr-*β*-CD, the Raman peaks of the Api and Que mixture were observed at 636, 714, and 757 cm^−1^, which are the characteristic peaks of Que. These peaks corresponded only to the characteristic peaks of Que. The results show that SiO_2_@Ag has potential for multiplex detection and that SiO_2_@Ag@pr-*β*-CD exhibits an ability to perform selective multiplex detection of specific targets.

**Figure 4:**
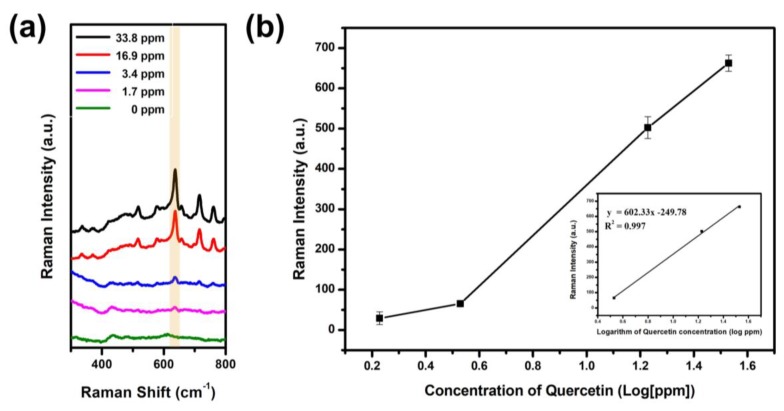
(**a**) Raman spectra of SiO_2_@Ag@pr-*β*-CD according to the concentration of Que and (**b**) its Raman intensity at 636 cm^−1^.

To investigate the dynamic linear range of Que, SERS spectra of SiO_2_@Ag@pr-*β*-CDs were treated by Que in the range of 1.69 to 33.8 ppm for 12 h. The result is shown in Figure 4. The Raman intensity of Que-incubated SiO_2_@Ag@pr-*β*-CD increased with the greater quantity of Que, particularly at the peak of 636 cm^−1^ (Figure 4a). Figure 4b shows the Raman intensities of SiO_2_@Ag@pr-*β*-CD, responding to the greater quantity of Que at 636 cm^−1^—i.e., the Raman intensity at 636 cm^−1^ increased proportionally with the increase in Que. The calibration curve of Que can be interpolated as a linear relationship between the Raman intensity and the logarithm of the Que concentration in the range of 3.4 to 33.8 ppm (y = 602.33 * log C − 249.78; R^2^ = 0.997, where C is the Que concentration) in the inset of Figure 4b. The limit of detection (LOD) of Que was determined to be 0.55 ppm (Figure S3), which is about 30 times more sensitive to the traditional Raman detection of Que (LOD of 15 ppm) [38]. The results suggest that the assembled structure with modified *β*-CD can enhance selectivity for capturing target molecules among similar structures by SERS.

## 4. Conclusions

We prepared pr-*β*-CD-functionalized SiO_2_@Ag (SiO_2_@Ag@pr-*β*-CD) for selective flavonoid detection. Both the SiO_2_@Ag@pr-*β*-CD and SiO_2_@Ag showed multiple detection potentials for flavonoids in SERS spectra, and the presence of pr-*β*-CD on the surface of SiO_2_@Ag was able to enhance the selectivity of the SERS probe. SiO_2_@Ag was able to detect all four flavonoids—Que, Myr, Api, and Nar—whereas SiO_2_@Ag@pr-*β*-CD was able to selectively detect Que and Myr. SiO_2_@Ag@pr-*β*-CD showed a dynamic linear range of Que from 3.4 to 33.8 ppm (R^2^ = 0.997), wherein Que was sdetectable at 0.55 ppm. We believe that our assembled structure with modified *β*-CD has successfully demonstrated selective capturing of certain targets amongst similar materials, which shows its considerable promise in immediate analyses of complex samples.

## Supplementary Materials

The following figures are available online at https://www.mdpi.com/2079-4991/9/10/1349/s1. Figure S1: ATR-FTIR spectra of SiO_2_@Ag, SiO_2_@Ag@pr-*β*-CD. These materials were measured in a solid state. Figure S2. UV-visible absorption spectra of SiO_2_@Ag@pr-*β*-CD-added flavonoids—quercetin (Que), myricetin (Myr), apigenin (Api) and naringenin (Nar). Figure S3. Normalized Raman intensity of SiO_2_@Ag@pr-*β*-CD according to the concentration of quercetin (Que) at 636 cm^−1^.
