# Supplementary material for: Mono-6-Deoxy-6-Aminopropylamino-β-Cyclodextrin on Ag-Embedded SiO2 Nanoparticle as a Selectively Capturing Ligand to Flavonoids
Source: Nanomaterials (Basel). 2019 Sep 20;9(10):1349. doi: 10.3390/nano9101349 (PMC6835478; doi:10.3390/nano9101349)
Supplement: Supplementary file 1 [file nanomaterials-09-01349-s001.pdf]

Supporting information

Mono-6-Deoxy-6-Aminopropylamino-*β*-Cyclodextrin on Ag-Embedded SiO_2 Nanoparticle as a Selectively Capturing Ligand to Flavonoids

Eunil Hahm [1], Eun Ji Kang [1], Xuan-Hung Pham [1], Daham Jeong [1], Dae Hong Jeong [2], Seunho Jung [1] and Bong-Hyun Jun [1,*]

[1] Department of Bioscience and Biotechnology, Konkuk University, Seoul 05029, Korea
[2] Department of Chemistry Education and Center for Educational Research, Seoul National University, Seoul 08826, Korea
* Correspondence: bjun@konkuk.ac.kr; Tel.: +82-2-450-0521

Received: 11 August 2019; Accepted: 17 September 2019; Published: date

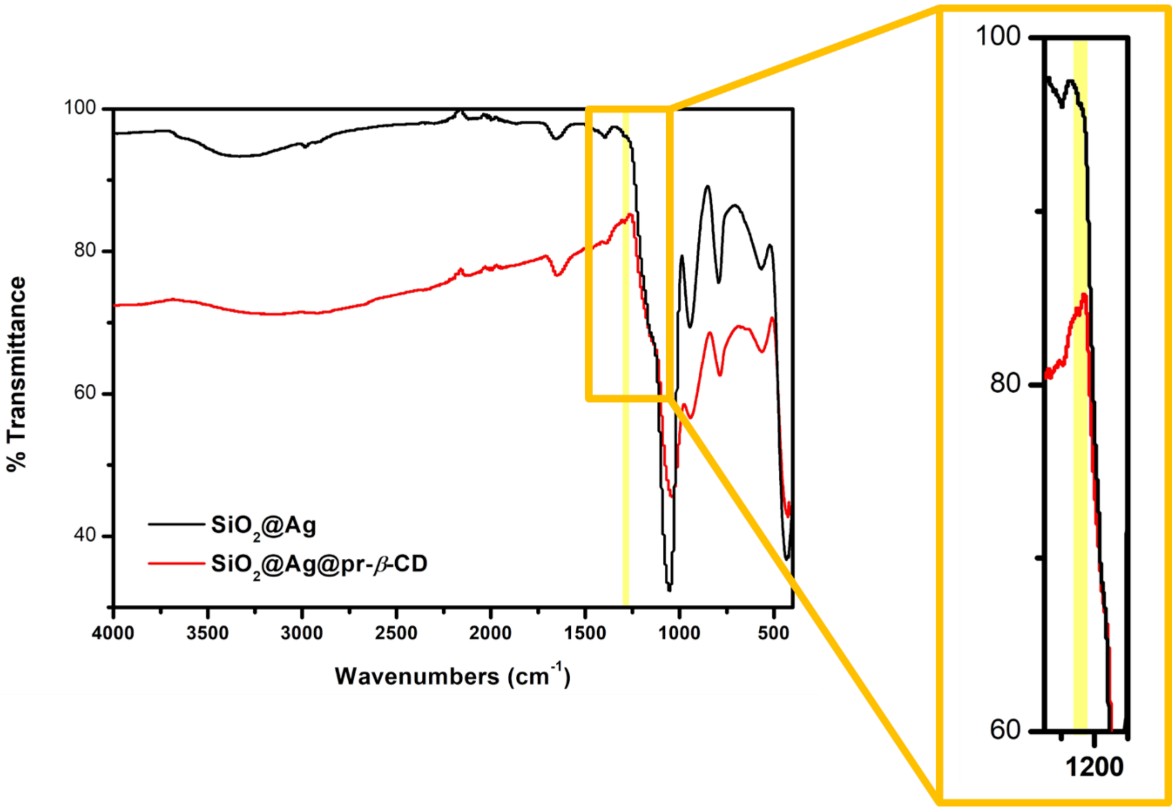

Figure S1. ATR-FTIR spectra of SiO_2@Ag, SiO_2@Ag@pr-*β*-CD. These materials were measured in a solid state.

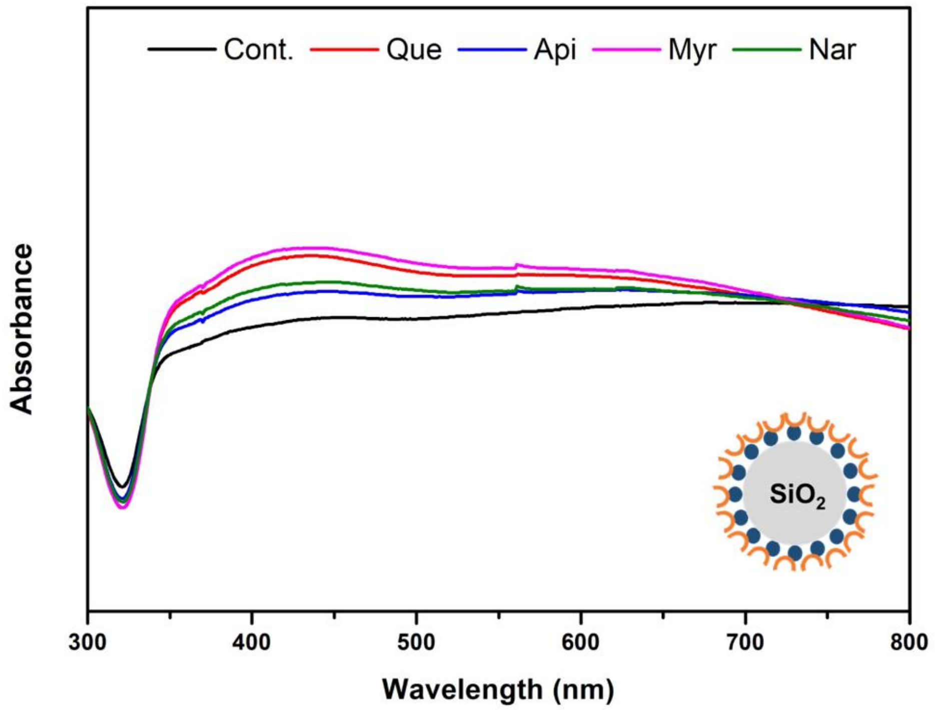

Figure S2. UV-visible absorption spectra of SiO_2@Ag@pr-β-CD-added flavonoids - quercetin (Que), myricetin (Myr), apigenin (Api) and naringenin (Nar).

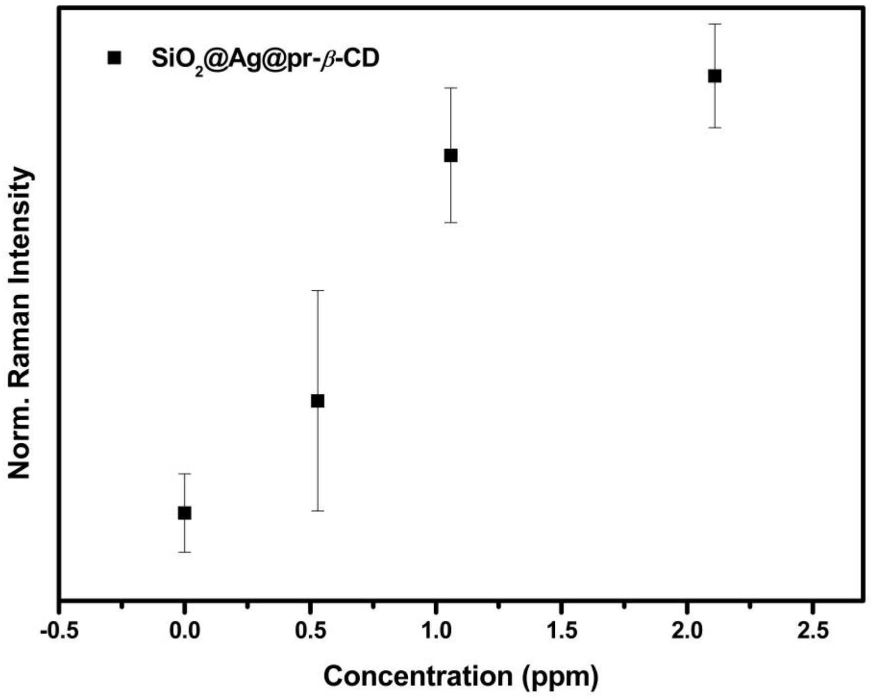

Figure S3. Normalized Raman intensity of SiO_2@Ag@pr-β-CD according to the concentration of quercetin (Que) at 636 cm^{-1}.
